# Extracting the GEMs: Genotype, Environment, and Microbiome Interactions Shaping Host Phenotypes

**DOI:** 10.3389/fmicb.2020.574053

**Published:** 2021-01-12

**Authors:** Ben O. Oyserman, Viviane Cordovez, Stalin Sarango Flores, Marcio F. A. Leite, Harm Nijveen, Marnix H. Medema, Jos M. Raaijmakers

**Affiliations:** ^1^Department of Microbial Ecology, Netherlands Institute of Ecology, Wageningen, Netherlands; ^2^Bioinformatics Group, Wageningen University & Research, Wageningen, Netherlands; ^3^Institute of Biology, Leiden University, Leiden, Netherlands

**Keywords:** microbiome, plant–microbe interactions, microbiome associated phenotype, microbial ecology, microbiome engineering, GEM

## Abstract

One of the fundamental tenets of biology is that the phenotype of an organism (*Y*) is determined by its genotype (*G*), the environment (*E*), and their interaction (*GE*). Quantitative phenotypes can then be modeled as *Y* = *G* + *E* + *GE* + *e*, where *e* is the biological variance. This simple and tractable model has long served as the basis for studies investigating the heritability of traits and decomposing the variability in fitness. The importance and contribution of microbe interactions to a given host phenotype is largely unclear, nor how this relates to the traditional GE model. Here we address this fundamental question and propose an expansion of the original model, referred to as GEM, which explicitly incorporates the contribution of the microbiome (*M*) to the host phenotype, while maintaining the simplicity and tractability of the original GE model. We show that by keeping host, environment, and microbiome as separate but interacting variables, the GEM model can capture the nuanced ecological interactions between these variables. Finally, we demonstrate with an *in vitro* experiment how the GEM model can be used to statistically disentangle the relative contributions of each component on specific host phenotypes.

## The Genetic Basis of Ecological Interactions

Leveraging the beneficial interactions between plant hosts and their microbiomes represents a new direction in sustainable crop production. In particular, the emergence of *m*icrobiome-*a*ssociated *p*henotypes (MAPs), such as growth promotion and disease suppression, is expected to reduce our dependency on energy-intensive and environmentally disturbing management practices. This may either be achieved through the addition of probiotics and prebiotics, or through breeding programs targeting MAPs to develop a next generation of “microbiome-activated” or “microbe-assisted” crop production systems (Busby et al., 2017; Oyserman et al., 2018). Hence, a major challenge is to identify the genotypic underpinning of emergent MAPs and understanding the pivotal role of the environment. The interaction between genotype (G) and environment (E) has long been recognized as an important factor both in evolutionary biology (Via and Lande, 1985; Anderson et al., 2013) and breeding programs (Allard and Bradshaw, 1964). While a significant body of literature exists on quantitative investigations of GE interactions (El-Soda et al., 2014), the bulk of this work has focused on abiotic parameters and has largely overlooked the microbiome. Nevertheless, the interactions between hosts, microbiomes, and their environments are coming into increasing focus and scrutiny (Dal Grande et al., 2018; Wallace et al., 2018; Beilsmith et al., 2019; Bonito et al., 2019). Indeed, researchers investigating pathogens often refer to the ‘disease triangle’ (Sandermann, 1996), whereas researchers investigating mycorrhizal–plant interactions often refer to the “context dependency” of inoculation success (Hoeksema et al., 2010), demonstrating a long history of investigations on GEM interactions. Consequently, as the prominence and importance of host-associated microbiome in modern biotechnology increases, it is important to explicitly integrate this variable into the widely accepted GE conceptual framework.

**FIGURE 1:**
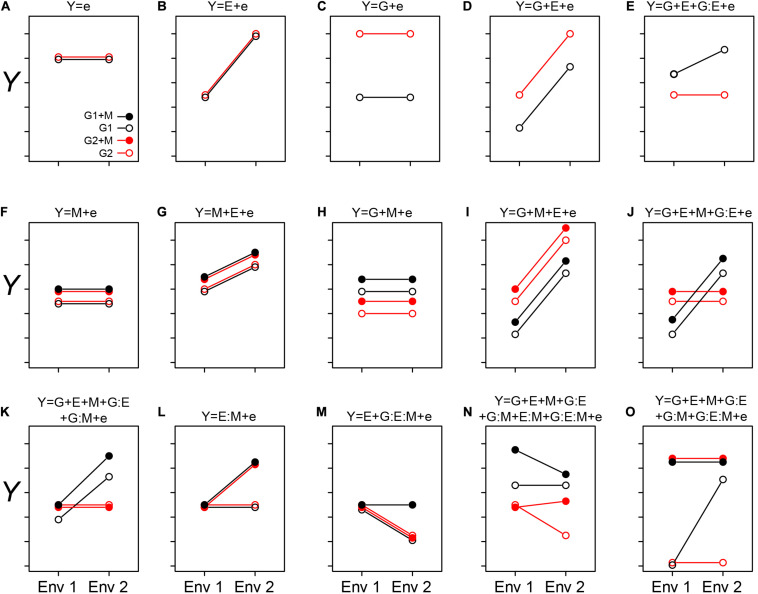
Conceptualizing the GEM model: Here we graphically explore how the interactions between genotypes, environment, and microbiome may impact a host phenotype (*Y*). The two genotypes are indicated by G1 and G2, and the presence of a microbiome is indicated by solid circles (as shown in **A**). The different environments are indicated as Env 1 and Env 2 on the *X*-axis. In each case **(A–O)**, the corresponding equation is depicted over the figure itself. In cases when we treat the microbiome as a phenotype of the host, the relative abundance of a particular taxon, or other features of a microbiome, may be considered as the sum of G and E interactions **(A–E)**. In simple cases, the relative abundance is independent of genotype **(B)** or environment **(C)**. More likely, both genotype and environment, and their interactions will contribute to relative abundance/function (**D** and **E**, respectively). **(A–E)** are special cases of the GEM model, indicating situations in which the microbiome does not contribute to a particular host phenotype. Building complexity, each of G, E, and M may contribute to host phenotypes individually or in combination, but without interaction **(A–D,F–I)**. Finally, the highest level of complexity occurs once interactions between G, E, and M occur **(E,J–O)**. A salient feature of this representation is that when no interaction between variables exists, the slope is equal between treatments. This model may also provide practical insights, such as identifying optimal prebiotics which may be expected to have a broad host range (no G interaction) and be conditionally neutral **(L)**. Additionally, this model may serve to characterize complex interactions, such as conditional symbiosis where a host fitness is reduced to zero without a microbiome (taxon or function) in a particular environment **(O)**.

**FIGURE 2:**
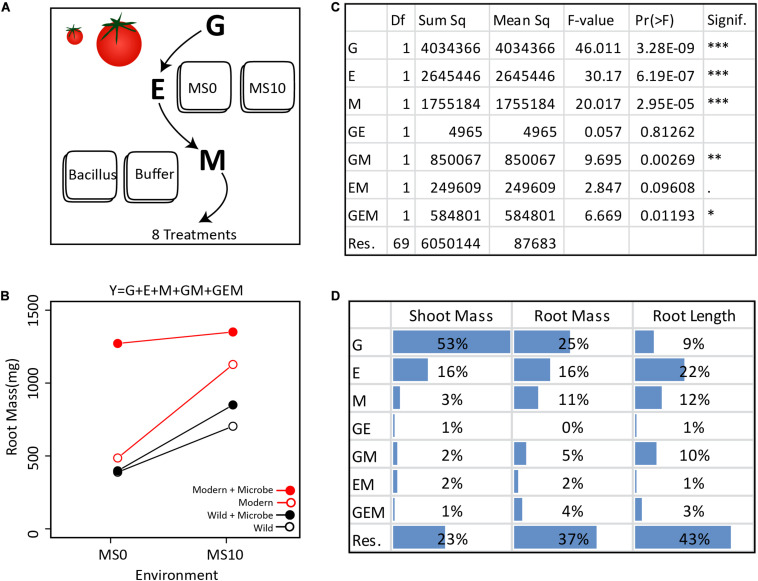
Extracting the GEMs from the simplified GEM experiment: **(A)** In this *in vitro* experiment, the contribution of G, E, M, and their interactions were investigated in a fully factorial design. **(B)** In total, two tomato genotypes, two environments, and one microbe treatment were investigated. Various plant phenotypes were measured, but for clarity, only the average dry root mass of each treatment are visualized here. **(C)** The GEM model shows that G, E, M, GM, and GEM all contribute significantly to root mass. The ANOVA table displays the reported Df (Degrees of freedom), Sum sq (Sum-of-squares), Mean sq (Mean some-of-squares), the F-value (the test statistic of an ANOVA), Pr(> F) (the *p*-value), and Signif. (a visual indication of the level of significance). **(D)** Here we present the ANOVA outcome showing the percent of the total sum of squares for dry shoot mass, dry root mass, and root length. For shoot mass, plant genotype explained the greatest portion of variance. In contrast, both E and M explained a greater amount of variation than plant genotype for root length. Importantly, for each of the three plant phenotypic parameters measured, GM explained a greater amount of variation than GE.

One current opinion is that rather than viewing host plants and animals as individuals, they should be viewed together with their microbiomes as single cohesive unit of selection termed a “holobiont” with a “hologenome” (Bordenstein and Theis, 2015; Moran and Sloan, 2015; Douglas and Werren, 2016). Under this view, the microbiome (M) could be integrated into the G term of the GE model of host phenotypes. However, others have pointed out that treating hosts and their microbiomes as a single unit does not capture the broad range of interactions and fidelity between host and microbe (Douglas and Werren, 2016). Another popular opinion is that, as the environment is classically defined to include “physical, chemical, and biotic factors (such as climate, soil, and living things) that act upon an organism” (Definition of Environment, 2019), M should be integrated into the E term of the GE model. However, an important distinction exists between E and M components; M is dynamic (i.e., have many interdependencies and may adapt or evolve through time), while E is driven through external processes. Here, we address these two viewpoints and propose that it is useful to introduce microbiomes and MAPs as a discrete unit within the GE model. In doing so, we put forth an updated GEM model that explicitly incorporates the microbiome (M) and its respective interactions with the genotype (G) and environment (E). Using these mathematical representations, we conceptually emphasize interesting cases that emerge from this framework (Figure 1). Next, we present a simple “one-microbe-at-a-time” experiment to highlight key features and challenges of unearthing GEM interactions, and to statistically disentangle the relative contributions of each of the GEM model components (Figure 2). Finally, we highlight the key challenges for moving forward in operationalizing such models effectively in complex natural systems.

## The Microbiome as a Phenotype or Microbiome-Associated Phenotypes?

The relationship between the host and its microbiome may be generally defined and viewed in two ways. First, microbiome community structure may be considered a phenotype of the host (*Y*), henceforth “microbiome as a phenotype” (Belheouane et al., 2017; Rothschild et al., 2018; Walters et al., 2018). Under this view, taxonomic/functional features of the microbiome are treated as the phenotype of the host (*Y*). In this manner, *Y* (e.g., the abundance of a taxon or functional gene) may be represented based on the contribution and interaction between the genotype (*G*), the environment (*E*), and the remaining variance (*e*) (Eq. 1). In extension, microbiome *(M)* components may also be included as predictive variables. For example, the successful establishment of rhizobia inoculants is often dependent on the abundance of indigenous rhizobia (Thilakarathna and Raizada, 2017), and the establishment of fungal pathogens may be dependent on the presence of arbuscular mycorrhizal fungi (AMF) (Berdeni et al., 2018). In these two examples, the abundance of beneficial inoculants or fungal pathogens may be treated as the phenotype of the host (*Y*) and modeled through the interactions of GE and M, where M is represented by the abundance of indigenous rhizobia and AMF, respectively.

Second, a microbiome may be quantified by their impact on the host phenotypes (Kopac and Klassen, 2016; Oyserman et al., 2018). In this view, MAPs such as plant growth promotion or plant tolerance to (a)biotic stress factors are treated as the phenotype (*Y*) (Zeevi et al., 2019). Here, we again suggest explicitly disentangling the environmental parameter of the traditional GE model (Eq. 1), such that host genotype (*G*), environmental factors (*E*), and microbiome structure and function (*M*) and their interactions all contribute to the observed host phenotype (Eq. 2). Thus, measurements of the microbiome structure and function are used in conjunction with genotypic and environmental data to explain a MAP, an emergent phenotype of the host–microbe interaction. Additional components may be added to the GEM model to accommodate additional complexity. For example, M may be split into *i* components, where M*_*i*_* represents the *i*th taxonomical or functional feature. In this way, the GEM model is amenable for investigating the role of microbe–microbe interactions within natural or synthetic communities, the interactions between multiple environmental factors, or any complex arrangements (see Supplementary Material for discussion on an expanded GEM model).

In Figure 1, we exhibit some basic features of the GEM model. In Figures 1A–E, quantitative microbiome features may be treated as a host phenotype (*Y*). Observed values of *Y* may be independent of changes in G and E (Figure 1A), dependent on E but not G (Figure 1B), dependent on G but not E (Figure 1C), dependent on G and E but not the interaction between GE (Figure 1D; the lack of an interaction is indicated by the equal slope of the two lines). Furthermore, *Y* may be dependent on both G and E and GE interactions. In Figures 1F–O, M may be integrated into the “microbiome as a phenotype” model (as in the examples with rhizobia and AMF above), or as a predictive variable of MAPs. In simple cases, M may not interact with either G or E (Figures 1F–J), but interactions between the various components of the GEM may also be observed (Figures 1K–O). By exploring this model, practical insights may be gleaned. For example, an optimal prebiotic would be conditionally neutral and have a broad host range (Figure 1L). Finally, the GEM model may be used to characterize complex interactions such as conditional symbiosis (Figure 1O), and in this manner captures a broad range of interactions and fidelity between host and microbe (Douglas and Werren, 2016).

As noted earlier, an important distinction between E and M is the dynamic nature of M. In other words, microbial populations may evolve to adapt to G, E, or GE interactions. Two simple illustrations of M adaptations to G were recently shown through the experimental evolution of *Aeromonas* for zebrafish colonization, and *Pseudomonas protegens* to *Arabidopsis thaliana* (Robinson et al., 2018; Li et al., 2020). In a reciprocal manner, M may precipitate adaptation in host G, as recently demonstrated in *Drosophila melanogaster* populations (Rudman et al., 2019). In this regard, the GEM model may be used to formulate and test hypotheses on how interactions drive evolutionary changes. From the “microbiome as a phenotype” perspective, *Y* would be considered the frequency of single nucleotide variants (SNV) or other marker of microbial population structure (Garud and Pollard, 2020; Yan et al., 2020). By using population genomics, the changes in SNV frequencies of natural microbial populations adapted to different host genotypes, and under specific conditions, may be reconstructed. Combining microbial population genetics with sufficiently large and genetically diverse host populations amenable to genome wide association studies (GWAS), it will be possible for future studies to map the reciprocal adaptions between host and microbe.

From the MAPs perspective, GEM interactions that result in the emergence of beneficial traits such as stress tolerance may lead to interesting eco-evolutionary dynamics. On the one hand, if the environmental conditions persist, directional selection may drive concerted fixation of host and microbe variants leading to coevolution (O’Brien et al., 2019). On the other hand, fluctuating selection driven by sufficient temporal or spatial heterogeneity may hamper the fixation of MAPs in a population, or over multiple generations. It also important to understand the mechanisms that maintain cooperation between host and microbiome and prevent the emergence of cheating phenotypes (Figueiredo and Kramer, 2020). For example, it has been shown that AMF and host use reciprocal rewards to stabilize beneficial interactions (Kiers et al., 2011). Thus, the rate (e.g., number of generations) at which host and microbiome may establish beneficial interactions (α_*holo*_), and the stability of these interactions (σ_*holo*_) within a host population or over subsequent generations are important parameters when investigating GEM interactions (Oyserman et al., 2018).

## Extracting the Gems

To demonstrate how the GEM model may be used to disentangle the relative influence of various factors on a particular host phenotype, we investigated GEM interactions in a simplified *in vitro* assay with one bacterial strain (*Bacillus* sp., accession number MN512243) interacting with two plant genotypes, a modern domesticated tomato cultivar (*Solanum lycopersicum* var moneymaker) and a wild tomato relative (*Solanum pimpinellifolium*) under two environmental conditions. In this model system, all genotype, environmental, and microbial parameters are controlled and therefore can be systematically explored in a fully factorial design (details are in the Supplementary Material). For each tomato genotype, seedlings were grown in two environments, i.e., Murashige and Skoog agar medium (MS0) and MS agar medium supplemented with 10 g/L of sucrose (MS10). After germination, the root tips were inoculated with the *Bacillus* strain, which was originally isolated from the wild tomato rhizosphere. Control seedlings were inoculated with buffer only (Figure 2A). The plant phenotypes monitored were root length (using WinRhizo^TM^) and root and shoot dry mass (Figure 2B). An ANOVA was done to test the significance of each variable in the GEM model (Figure 2C). Together, the microbiome (M) and all interacting variables (GM, EM, and GEM) explained 22% of root dry mass variance, 8% of shoot dry mass variance, and 26% of root length total variance. Furthermore, in all cases, the interacting parameters, GM, EM, and GEM interactions explained greater variance than GE interactions (Figure 2D).

**EQUATION 1:**
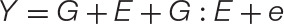
The traditional model for GE interactions: In the canonical model of quantitative phenotypes, the host phenotype (*Y*) is explained by the sum of G, E, their interactions (G:E), and e the residual error. This model may be used to calculate the proportion of variance explained by the host genome and the environment on a host associated microbiome community. In other words, the microbiome may be treated as *Y*, the phenotype of the host (e.g., “the microbiome as a phenotype”). When E has no contribution to *Y*, only G determines the abundance or function of the microbiome (Figure 1C). On the other side of the spectrum, only E determines to the abundance or function of the microbiome (Figure 1B).

**EQUATION 2:**
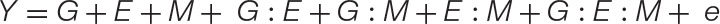
The new GEM model: When a microbiome has a quantitative impact on host phenotype, the traditional GE model may be expanded to incorporate M and all respective interactions (GM, EM, and GEM). Unlike the GE model, which may be used to explain the microbiome, the expanded GEM model may be used to disentangle the contribution of G, E, and M and their various interactions to changes in host phenotype. When M has no impact, this variable and those associated with it fall out of the equation giving the GE model. These and other special cases are conceptually explored further in Figure 2. Thus, this model is capable of capturing the nuanced dynamics of host–microbiome interactions, such as host–microbe interactions that are environment-specific, or otherwise have lower fidelity than strict symbiosis (Douglas and Werren, 2016).

**EQUATION 3:**
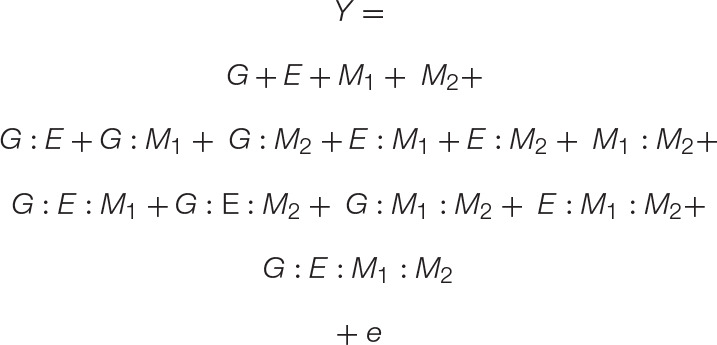
A GEMM model: The basic GEM model may be expanded to include any number of complex interactions. Here we expand the GEM model to include microbe–microbe interactions. This results in the addition of one-way, two-way, three-way, and four-way interaction terms, which are shown on separate lines for clarity.

A clear consensus is forming that microbiomes impact host phenotypes, but its relative contribution to that host phenotype is, in most cases, not known. The GEM model provides a simple, tractable, and testable model demonstrating that the interactions of the microbiome and other model terms (GM, EM, and GEM) are also essential determinants of host phenotypes. It is important to highlight that, in this case, GM interactions actually explain more variability than canonical GE interactions. Furthermore, the expanded GEM model captures other important features that may otherwise be easily overlooked, such as the genotype-independent interaction between EM. This states that microbe and environment may interact to alter host fitness independent of the genotype. For example, pre-conditioning soil microbial populations to drought has been shown to select for microbial communities which promote host drought tolerance when compared with un-conditioned naive soils (Lau and Lennon, 2012). Additionally, auxin is a plant hormone that promotes growth that is also produced by bacteria. Many bacterial cultures have differential auxin production dependent on their environment (Tsavkelova et al., 2005); therefore, it is likely that EM interactions can promote auxin production and thus plant growth independent on genotype. In practice, identifying EM may have important implications for synbiotics (mixtures of probiotics and prebiotics) and the development of self-assembled microbiomes (Gutierrez et al., 2020). In this manner, the GEM model not only provides a model to disentangle the contribution of G, E, and M, but also serves as a powerful tool for conceptualization and experimental design.

## The Gem Model Parameterizes Complex Interactions

As described above, genotype, environment, and microbiome may influence organismal phenotype directly, but also through their interactions. This dynamic is captured by the various *terms* that make up the GEM model, providing a simple means to parameterize an otherwise complex system. In its most basic form (Eq. 2), the GEM model has eight terms in total. An example of a term with a single variable is “G,” a two-variable term would be “GM,” and three variable term would be “GEM.” While the basic GEM model contains terms related to inter-class interactions (GE, GM, etc.), it lacks terms representative of intra-class interactions (M:M, E:E, etc.). By simply adding additional variables to the GEM model, M:M and other ecologically relevant interactions may be introduced as additional terms. The number of terms in a model is dependent on the number of variables (*n*) that can be mathematically represented by Supplementary Equation 1. In addition, the number of terms with *r* variables may be mathematically represented by Supplementary Equation 2, where *n* is the total number of variables and *r* is the number of variables in the term. From this basis, a model of organismal phenotype which takes into account ecosystem-level processes may be constructed. To this end, we developed a simple Python script to generate a GEM model based on user input for any number of G, E, and M variables^1^.

To model the interactions between multiple microbiome members, such as those found in natural or synthetic communities, we provide a simple expansion in Eq. 3. The result is a four-variable (GEM_1_M_2_) model that includes all *r*-way interactions terms necessary to model the impact of a two-member community on any number of plant genotypes or environments. For clarity, Eq. 3 is presented with all *r*-way interactions on separate lines. To show the versatility of the GEM model, we provide another expansion in which multiple hosts are interacting in a particular ecosystem (G_1_G_2_EM). In this case, the fitness of one plant genotype (G_1_) is influenced through interactions with a neighboring plant genotype (G_2_) and their associated microbiomes. A prominent example of this in literature are intercropping systems in which nitrogen fixation through legume–microbiome interactions benefit other non-leguminous plants in a nitrogen limited soil ecosystem (Peoples et al., 1995). Indeed, the literature is filled with examples that fit the GEM model, including interactions involving mycorrhizal fungi (Hoeksema et al., 2010), rhizobia (Lau et al., 2012), endophytes (Zhou et al., 2019), and concerning a variety of emergent phenotypes from diverse interactions (DeMilto et al., 2017).

While the GEM model provides a simple conceptual framework for understanding the microbiome contribution to host phenotype, a key challenge will be incorporating complex natural microbiomes containing hundreds of species and thousands of interactions in natural settings. In addition, it is likely that observational studies on GEM interactions may be further hampered by covariance between microbiomes, host genotype, and the environment. Altogether, a proper statistical approach to handle GEM model should account for: (i) the different data characteristics and sources; (ii) the co-dependence structure between and within groups of variables; (iii) the specific effect of each component (genes, microbes, and environment) on the plant phenotype. To date, few methods can capture this complexity. A promising approach is via generalized joint attribute modeling (GJAM) (Clark et al., 2017; Leite and Kuramae, 2020). GJAM allow us to infer and interpret relationship between different groups of variables (e.g., continuous such as plant biomass, or compositional as the DNA copy number) on the observation scale and to avoid distorted correlations. For example, GJAM was recently applied to identify 12 AMF associated with less foliar damage in seedlings from different plant species in mid- and late-successional subtropical montane forests in Puerto Rico (Bachelot et al., 2018). Therefore, GJAM combines environmental factors and microbiome data with the plant phenotype into a single framework. However, through careful experimental design and reductionist approaches, it is likely that the coming years will see rapid headway identifying genes responsible for recruiting microbes (i.e., the microbiome as a phenotype), and identifying the genes underlying the emergent phenotypes from plant–microbe interactions (i.e., microbiome associated phenotypes).

## Conclusion

A fundamental tenet of biology is that genotype and environment interact and impact the fitness and phenotype of an organism. The GE model of organismal phenotype has been the cornerstone of modern breeding programs. Part of the power of the GE model is its simplicity and interpretability. However, the important role of host-associated microbiomes has recently come into focus. Here, we investigated how microbiomes (M) fit into the GE model, suggest an explicit expansion to include M, and argue that, because of its dynamic and evolving nature, that M should not be collapsed within E. We use a conceptual figure to show that the updated GEM model captures the diverse possible outcomes of between G, E, and M. To support our model, we present an *in vitro* experiment with one microbe demonstrating not only how to use the GEM model, but also showing that GM interactions may explain more variability than GE interactions. Finally, additional examples of expanded GEM models which take into account M:M and G_2__:_E:M interactions are presented to demonstrate the ecological versatility of the GEM model. Taken together, we propose that the GEM model provides a simple and interpretable expansion of the GE model. Furthermore, given the important role of the microbiome, any investigations into GE interactions must also account or control for M.

## Data Availability Statement

The datasets presented in this study can be found in online repositories. The names of the repository/repositories and accession number(s) can be found in the article/Supplementary Material.

## Author Contributions

The ideas presented here were conceived through discussion and interaction between all authors. BO, SF, and VC performed the experiments. BO wrote the manuscript. All authors discussed the results, provided feedback during the writing process, and commented on the final manuscript.

## Conflict of Interest

The authors declare that the research was conducted in the absence of any commercial or financial relationships that could be construed as a potential conflict of interest.

## Footnotes

1 https://github.com/Oyserman/GEM
