# Supplementary material for: Extracting the GEMs: Genotype, Environment, and Microbiome Interactions Shaping Host Phenotypes
Source: Front Microbiol. 2021 Jan 12;11:574053. doi: 10.3389/fmicb.2020.574053 (PMC7874016; doi:10.3389/fmicb.2020.574053)
Supplement: Supplementary file 2 [file Data_Sheet_2.docx]

**Supplemental Materials**

# Extracting the GEMs: Genotype, Environment and Microbiome interactions shaping host phenotypes

Ben O. Oyserman^1,2^, Viviane Cordovez^1,3^, Stalin Sarango Flores^3^, Marcio F. A. Leite^1^, Harm Nijveen^2^, Marnix H. Medema^2^, Jos M. Raaijmakers^1,3^

1 Department of Microbial Ecology, Netherlands Institute of Ecology, 6708 PB Wageningen, The Netherlands

2 Bioinformatics Group, Wageningen University & Research, 6708 PB Wageningen, The Netherlands

3 Institute of Biology, Leiden University, 2333 BE Leiden, The Netherlands

**Supplemental Methods and Results**

The plants were grown in a Snijder Labs (Tilburg NL) Micro Clima-Series^TM^ High Specs Plant Growth Chamber under the following conditions: 21 °C, 70 % humidity, 16hr/8hr day/night cycle, and illumination levels of 60 TL and 30 LED. Two *in vitro* environments were tested. The first environment was a half strength Murashige and Skoog medium (MS, Duchefa Biochemie B.V.) with vitamins, 1.2 % plant agar and with pH 5.8 adjusted with potassium hydroxide. The second environment was the same media but supplemented with 10 g/L of sucrose. These two environments are referred to as MS0 and MS10 in the manuscript, indicating the absence and presence of sugar respectively. Each 12.5–cm-diameter square petri dish received 50 mL of media.

The modern tomato plant *Solanum lycopersicum* (var Moneymaker) and its wild relative *Solanum pimpinellifolium* were used in this experiment. Before sowing, the seeds were surface sterilized in 70 % ethanol for 2 minutes followed by 2.5 % bleach for 20 minutes. Both wash solutions were prepared with sterile demi water. Seeds were then triple rinsed with sterile water. Sterile seeds were placed on sterile wet filter paper placed in a 9-cm-diameter petri dish and kept at 25 °C in the dark for 4 days. Germinated seeds were place on petri dishes containing MS0 or MS10. For each treatment, 3 seeds were placed equidistant to each other in a single line 3.5 cm below the top of a 12.5 cm square petri dish. A total of 10 replicates were prepared for each treatment. Agar plates were sealed and then placed at a slight angle in growth chamber and grown until harvest. Harvesting was done once the first set of true leaves had fully emerged for the fastest growing treatment of each genotype. Modern and wild seedlings were harvested 12 and 13 days after sowing, respectively.

Each treatment was inoculated with either sterile 10 mM MgSO_4_ (hereafter: buffer) or a microbial inoculant. For the microbial inoculant, a *Bacillus* sp. strain PRA10 originally isolated from a wild tomato rhizosphere was used. A BLAST analysis of the partial (834 bp) 16S rDNA sequence retrieved from this isolate shows that it shares 100% coverage and identify with *Bacillus paramycoides*, *B. albus* and *B. cereus*. Briefly, the *Bacillus* strain was previously grown in Tryptone Soy Agar (TSA, Oxoid) at pH 7 at 25 °C for 4 days. Bacterial cells were re-suspended in the buffer at an optical density (OD_600_) of 1 before inoculation. Plant root tips were inoculated with 2 uL of the bacterial solution. All work was done in a sterile environment. A ‘root imprinting’ assay was conducted on an extra replicate from each treatment to confirm the root colonization by the strain. Root imprinting was done by overlaying the tomato root on TSA (pH 7) for a few seconds and then removing the plant and incubating for 4 days. Visual observation confirmed the colonization and presence of a single isolate.

To investigate the impact of each treatment on phenotype, shoot and root dry weight as well as root architecture parameters were analyzed. For each replicate, the shoots were cut from the roots and bagged in a pooled sample per replicate. Next, the plates containing the roots were scanned and analyzed with WinRHIZO^TM^. After scanning, the roots were harvested and bagged similarly in pooled samples per replicate. Shoot and roots were dried at 60 °C and weighed. The full results from the dry weights and the root architecture parameters can be found in Supplemental Table 1 and 2 respectively.

$$2^{n}-1$$

Supplemental Equation 1. The total number of terms in a GEM model is dependent on the number of components in the model (n).

$$\frac{n!}{(r!(n-r)!)}$$

Supplemental Equation 2. The number of terms with r variables may be mathematically represented, where n is the total number of variables, and r is the number of variables in the term.
